# Comprehensive Anatomical Staging Predicts Clinical Progression in Mild Cognitive Impairment: A Data-Driven Approach

**DOI:** 10.3390/ijms26125514

**Published:** 2025-06-09

**Authors:** Raghav Tandon, Yajun Mei, James J. Lah, Cassie S. Mitchell

**Affiliations:** 1 Laboratory for Pathology Dynamics, Department of Biomedical Engineering, Georgia Institute of Technology and Emory University School of Medicine, Atlanta, GA 30332, USA; 2 Center for Machine Learning, Georgia Institute of Technology, Atlanta, GA 30332, USA; 3 Department of Biostatistics, School of Global Public Health, New York University, New York, NY 10003, USA; yajun.mei@nyu.edu; 4 Alzheimer’s Disease Research Center, Department of Neurology, Emory University School of Medicine, Atlanta, GA 30329, USA; jlah@emory.edu

**Keywords:** Alzheimer’s disease progression, mild cognitive impairment, disease heterogeneity, cognitive decline prediction, neurodegeneration, artificial intelligence, behavioral neurology, aging

## Abstract

Alzheimer’s disease (AD) presents significant challenges in clinical practice due to its heterogeneous manifestation and variable progression rates. This work develops a comprehensive anatomical staging framework to predict progression from mild cognitive impairment (MCI) to AD. Using the ADNI database, the scalable Subtype and Stage Inference (s-SuStaIn) model was applied to 118 neuroanatomical features from cognitively normal (*n* = 504) and AD (*n* = 346) participants. The framework was validated on 808 MCI participants through associations with clinical progression, CSF and FDG-PET biomarkers, and neuropsychiatric measures, while adjusting for common confounders (age, gender, education, and APOE ε4 alleles). The framework demonstrated superior prognostic accuracy compared to traditional risk assessment (C-index = 0.73 vs. 0.62). Four distinct disease subtypes showed differential progression rates, biomarker profiles (FDG-PET and CSF Aβ42), and cognitive trajectories: Subtype 1, subcortical-first pattern; Subtype 2, executive–cortical pattern; Subtype 3, disconnection pattern; and Subtype 4, frontal–executive pattern. Stage-dependent changes revealed systematic deterioration across diverse cognitive domains, particularly in learning acquisition, visuospatial processing, and functional abilities. This data-driven approach captures clinically meaningful disease heterogeneity and improves prognostication in MCI, potentially enabling more personalized therapeutic strategies and clinical trial design.

## 1. Introduction

Alzheimer’s disease (AD) poses challenges in clinical practice due to its heterogeneous manifestations and variable progression [1,2]. Predicting progression from mild cognitive impairment (MCI) to AD is crucial, as this transition represents a key window for intervention [3,4]. While risk factors such as age, APOE ε4 status, and cognitive scores provide prognostic value, their utility in individualized care is limited due to the complex nature of disease progression [5]. Progression follows a trajectory from a preclinical state to MCI and, ultimately, AD, emphasizing the importance of early diagnosis and treatment.

AD pathology accumulates during a lengthy preclinical period before clinical symptoms appear [6]. Early intervention during the MCI stage is particularly critical, as emerging treatments may be most effective in this phase. Reliable tools to identify and monitor progression in MCI patients are increasingly important for both clinical practice and trial design.

Neuroimaging, particularly structural MRI, has emerged as a valuable tool in AD assessment, offering insights into patterns of neurodegeneration [7]. While current applications typically focus on specific regions known to be affected early in disease progression, mounting evidence suggests that AD-related changes occur across multiple brain regions [8,9,10]. For example, while typical AD primarily affects memory, some patients show visual, language, or executive deficits, reflecting distinct neurodegeneration profiles [11].

Recent computational advances enable a comprehensive analysis of neuroanatomical changes. Event-based models (EBMs) capture disease trajectories using cross-sectional and multimodal data [12,13,14]. However, many focus on limited brain regions or ignore disease heterogeneity, thereby missing critical prognostic details. This study applies the scaled Subtype and Stage Inference (s-SuStaIn) algorithm [15] to analyze brain atrophy patterns in AD. Using Alzheimer’s Disease Neuroimaging Initiative (ADNI) data, this study evaluates the framework’s ability to predict MCI-to-AD progression while considering disease heterogeneity. The study’s objectives include (1) developing a disease staging and subtyping framework using MRI measures, (2) evaluating its prognostic value, and (3) linking it to established clinical, cognitive, and biological markers of disease progression. This work aims to enhance personalized prognostication, improving patient counseling and clinical trial stratification.

## 2. Results

### 2.1. MCI to AD Conversion Risk: Stage, Subtype, and Neuroanatomy

**Figure 1:**
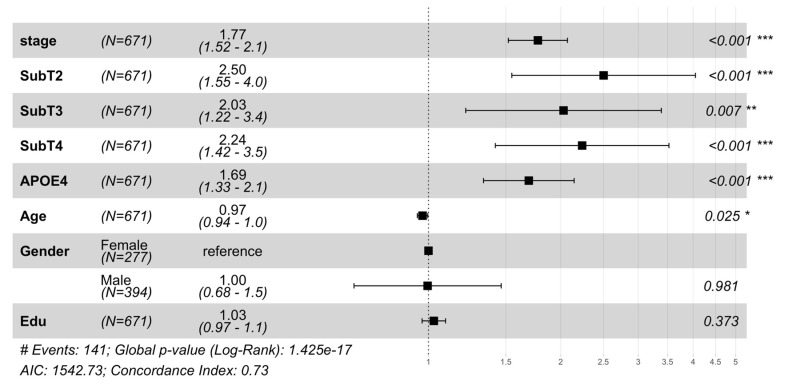
Hazard ratios from the Cox proportional hazards (Cox PH) model were used to analyze progression from mild cognitive impairment (MCI) to Alzheimer’s Disease dementia in the validation cohort. The plot shows hazard ratios with 95% confidence intervals for disease stage, subtype, APOE4 status, and demographic variables (age, gender, and education). Subtype 1 (SubT1) serves as the reference group for subtype comparisons (SubT2, SubT3, and SubT4 denote Subtypes 2, 3, and 4, respectively), and female gender serves as the reference for gender comparison. Hazard ratios > 1 indicate increased risk of conversion to dementia. s-SuStaIn inferred disease stages and subtypes showed significant associations (*p* < 0.05) with conversion risk to AD while adjusting for important demographic (age, gender, and education) and genotypic (APOE ε4) risk factors. The model demonstrated good predictive performance (concordance index (C-Index) = 0.73, Akaike Information Criterion (AIC) = 1542.73) and significantly outperformed a reduced model containing only demographic and genetic variables (concordance index = 0.62, AIC = 1604.39) and log-rank *p*-value (1.42 × 10^−17^ vs. 1.3 × 10^−5^), hence demonstrating the improvement in overall survival modeling by including s-SuStaIn-inferred stages and subtypes. * (*p* < 0.05), ** (*p* < 0.01), *** (*p* < 0.001).

MCI-to-AD progression risk was analyzed using a Cox proportional hazards model in the validation cohort (671/808 MCI subjects with follow-up data), shown in Figure 1. The model incorporating s-SuStaIn measures (stage and subtype) achieved a concordance index (C-index) of 0.73 and AIC of 1542.73, outperforming traditional models based on demographics and genetics (C-index = 0.62, AIC = 1604.39).

Disease stage was observed to be a strong predictor of progression (HR = 1.77, *p* = 3.8 × 10^−13^). Subtype-specific risks were significant compared to Subtype 1 (SubT1): SubT2 (HR = 2.50, *p* = 1.8 × 10^−4^), followed by SubT4 (HR = 2.24, *p* = 5.2 × 10^−4^), and SubT3 (HR = 2.03, *p* = 0.007). Subtype heterogeneity in progression risk and age of onset is shown in Figures S1 and S2. A t-SNE projection of neuroanatomical features in MCI subjects highlights variations by subtype and stage (Figure S3). These results highlight the significance of inferred stages and subtypes in capturing the heterogeneous progression from MCI to AD. Subtype-specific progression patterns were tested across eight regional brain configurations (34–118 neuroanatomical features, Table S1). Although the disease stage predicted progression across all configurations, subtype-specific risks emerged only when using all 118 neuroanatomical features, suggesting that comprehensive anatomical profiling better captures the heterogeneity of the disease, aligning with previous work [16,17,18].

### 2.2. Progression of AD Biomarkers and Brain Atrophy

#### 2.2.1. Glucose Metabolism and Amyloid Pathology Subtype-Specific Patterns

**Figure 2:**
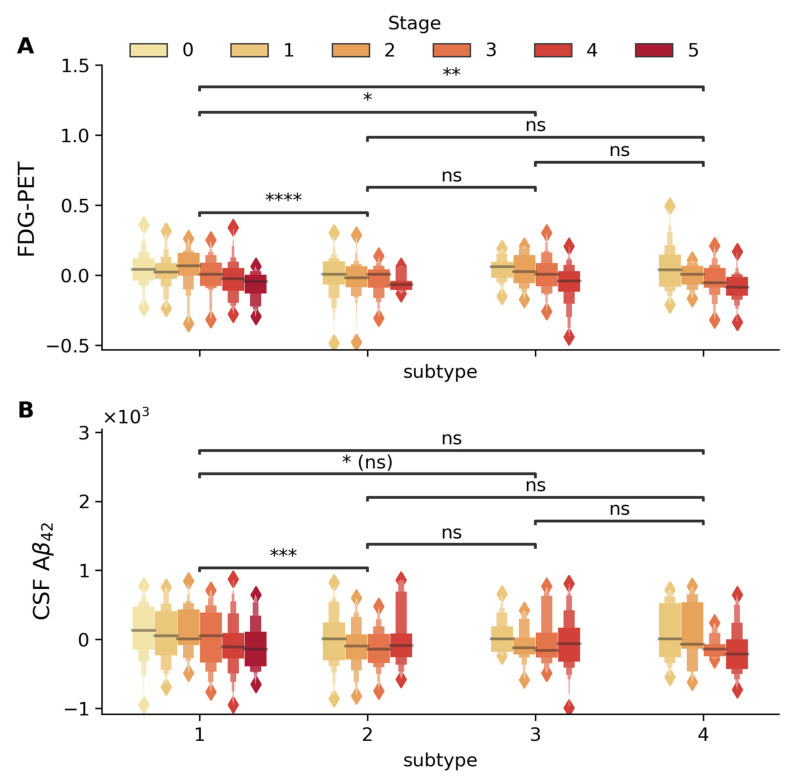
Distribution of FDG-PET (standard uptake value) and CSF Aβ_42_ (pg/mL) biomarkers across disease stages and subtypes. Box plots display the stage-wise progression (stages 0–5, shown in color gradient from light to dark) within each subtype. (**A**) FDG-PET and (**B**) CSF Aβ_42_ within each subtype (1–4). Statistical significance of between-subtype comparisons is indicated by horizontal bars (* *p* < 5 × 10^−2^, ** *p* < 5 × 10^−3^, *** *p* < 5 × 10^−4^, **** *p* < 5 × 10^−5^, ns: not significant). FDG-PET metabolism shows significant differences between Subtypes 1 and 2, 1 and 3, and 1 and 4, with significant stage-wise decline in Subtypes 1 (β_stage_ = −0.021 *p* = 7.6 × 10^−7^), 3 (β_stage_ = −0.035, *p* = 1.2 × 10^−3^), and 4 (β_stage_ = −0.044, *p* = 7.9 × 10^−5^). CSF Aβ_42_ levels demonstrate significant variation between Subtypes 1 and 2, with progressive stage-wise reduction particularly evident in Subtypes 1 (β_stage_ = −46.34, *p* = 9.3 × 10^−4^) and 4 (β_stage_ = −88.18, *p* = 8.2 × 10^−3^). All analyses were adjusted for age, gender, education, and APOE4 status, with between-subtype comparisons additionally adjusted for disease stage.

Two key biomarkers validated subtypes in baseline MCI patients (Figure 2), FDG-PET metabolism (average FDG-PET of angular, temporal, and posterior cingulate) and CSF Aβ_42_ level, showed significant differences across subtypes (adjusted for demographic and genetic confounders). Subtype 1 showed marked differences for FDG-PET and CSF Aβ_42_ levels compared to other subtypes (e.g., Subtype 1 vs. 2, Mann–Whitney–Wilcoxon *p* < 5 × 10^−5^ in both cases, FDR-corrected). Both biomarkers also declined progressively with advancing stages in specific subtypes (β_stage_ = −0.035, *p* = 4.45 × 10^−17^ for FDG-PET, β_stage_ = −63.93, *p* = 8.3 × 10^−7^ for CSF Aβ_42_). Both biomarkers also declined progressively with advancing stages, particularly in subtype 1 (β_stage_ = −0.021, *p* = 7.6 × 10^−7^ for FDG-PET and β_stage_ = −46.34, *p* = 9.3 × 10^−4^ for CSF Aβ_42_).

Including subtype and stage improved variance explained for glucose metabolism (adj. R^2^ improved from 10.4% to 20.6%) and CSF Aβ_42_ (adj. R^2^ from 24.8% to 28.4%), capturing heterogeneity beyond traditional risk factors. Past work in [17,19] has also shown that disease progression and heterogeneity are captured by FDG-PET and CSF Aβ_42_ levels.

#### 2.2.2. Regional Brain Volume Changes

**Figure 3:**
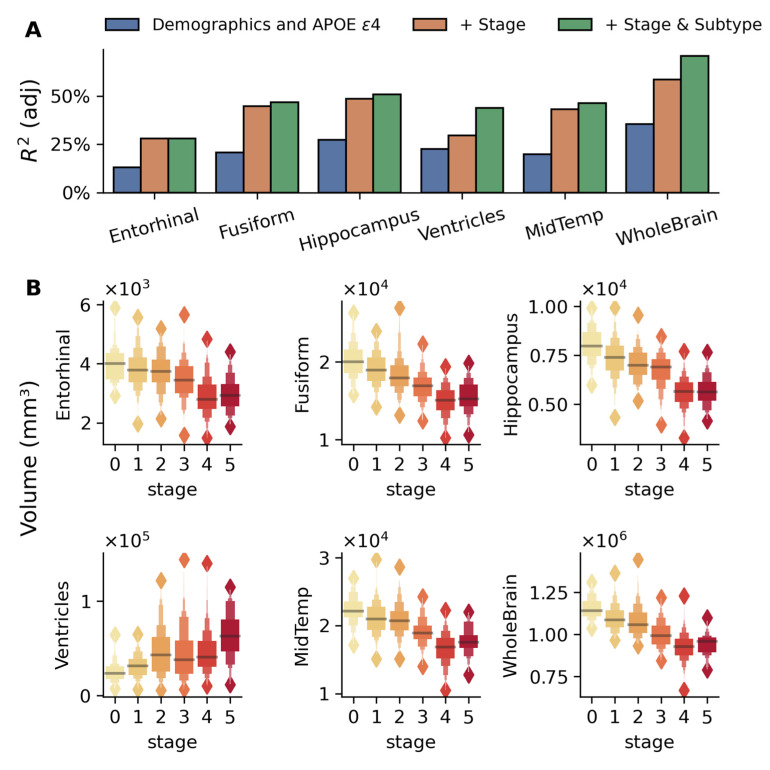
Brain regional volumes explained by the model-inferred stages and subtypes in the MCI validation cohort. (**A**) Bar plots compare adjusted R^2^ values from three nested linear regression models for each brain region: baseline model with demographics (age, gender, and education) and number of APOE ε4 alleles (blue), model adding disease stage (orange), and full model including both stage and subtype (green). The sequential improvement in R^2^ values demonstrates the utility of disease stage and subtype beyond traditional risk factors, with particularly strong improvements seen in the whole-brain, hippocampus, and fusiform regions. (**B**) Box plots show the distribution of volumes across disease stages (0–5, indicated by color gradient) for six brain regions: the entorhinal cortex, fusiform gyrus, middle temporal gyrus (MidTemp), ventricles, hippocampus, and whole brain. The association between these volumes and disease stages is highly significant (*p* < 10^−28^ for all regions) while adjusting for age, gender, education, APOE ε4 alleles, and subtypes. Progressive atrophy is evident in all regions except the ventricles, which show expansion with disease progression. Values are presented in cubic millimeters (mm^3^).

Structural MRI analysis using sequential nested regression models (Figure 3A) demonstrated increasing explanatory power across brain regions. The base model with demographics and APOE ε4 alone explained 13–35% variance in brain regional volumes, with stage inclusion improving adj. R^2^ to 30–60%, and subtype addition further increasing adj. R^2^ for whole-brain (70%) and ventricular volumes (44%). These volumetric changes were significant across disease stages in key AD-related regions (*p* < 10^−28^, for all regions shown in Figure 3), controlling for demographics, APOE4, and subtypes (Figure 3B). These results suggest that the progressive brain volumetric changes across AD are best explained when considering both disease stage and subtypes.

### 2.3. Stage-Dependent Changes in Cognitive Performance

#### 2.3.1. Global Cognitive Measures

**Figure 4:**
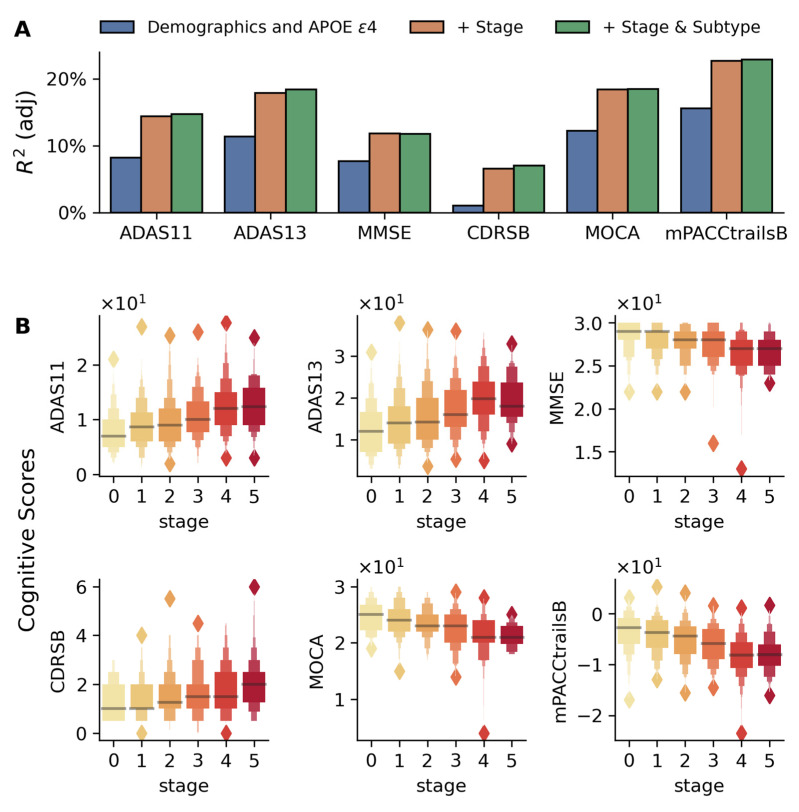
Association between disease stages and cognitive test scores in the validation cohort. (**A**) Bar plots comparing adjusted R^2^ values from three nested linear regression models for each cognitive measure: baseline model with demographics and APOE ε4 (blue), model including disease stage (orange), and full model with both stage and subtype (green). The progressive increase in adjusted R^2^ values demonstrates the importance of incorporating disease stages and subtypes in predicting cognitive performance. (**B**) Box plots show the distribution of six cognitive measures (CDR-SB, ADAS11, ADAS13, MOCA, MMSE, and modified PACC) across disease stages (0–5, shown in color gradient from light to dark). The association between cognitive test scores and inferred stages is adjusted for age, gender, education, APOE ε4 alleles, and disease subtypes. In each case, a highly significant association is found between the disease stage and cognitive scores (*p* < 5 × 10^−8^ for all cases).

Cognitive and clinical measures (e.g., Alzheimer’s Disease Assessment Scales (ADAS11, ADAS13) and Clinical Dementia Rating–Sum of Boxes (CDR-SB)) showed significant associations with disease stage (Figure 4). Adding the stage improved model R^2^ substantially (e.g., ADAS13: 11.3% to 18.4%, Figure 4A). ADAS scores distinguished early stages (e.g., stage 0 vs. 1, * *p* < 0.05), while MMSE and mPACCtrailsB differentiated advanced stages (** *p* < 5 × 10^−4^), as shown in Figure S4.

Composite scores for memory, executive function, language, and visuospatial abilities (proposed in [20,21]) also showed strong associations with disease stage (Figure S5), demonstrating cognitive decline over multiple domains and its association with the inferred disease progression trajectory. In each case, adjustments were made for demographics, APOE ε4, and subtype.

#### 2.3.2. Learning and Memory Tests

**Figure 5:**
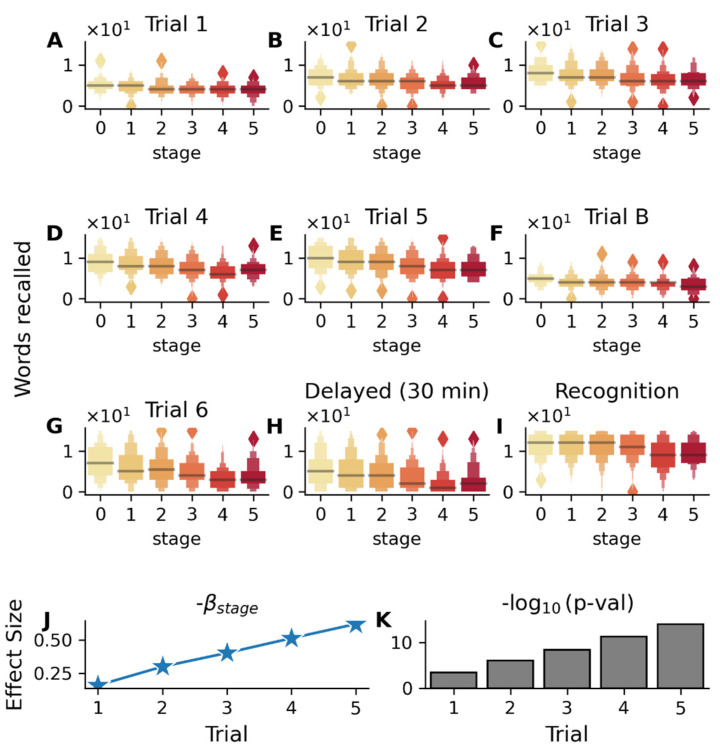
Association between RAVLT (Rey Auditory Verbal Learning Test) performance and disease stages in the validation cohort (MCI subjects). Color gradient from light to dark in (**A**–**I**) represents increasing disease stages. (**A**–**E**) Box plots showing the distribution of scores (number of words recalled in each trial) across disease stages (0–5) for immediate recall trials (1–5), (**F**) interference list (Trial B), (**G**) post-interference recall (Trial 6), (**H**) delayed recall (30 min), and (**I**) recognition memory. The association between test scores and s-SuStaIn inferred disease stages is adjusted for age, gender, education, APOE ε4 status, and disease subtypes. (**J**) Analysis of learning Trials 1–5 showing increasing magnitude of β_stage_ coefficients and (**K**) strengthening statistical significance (−log_10_(*p*-value)) across successive learning trials 1-5, indicating that subjects in earlier disease stages become better at recall with increasing trials, while subjects in the later stages do not improve as much. Significant association with disease stage is also seen for the interference list (Trial B, β_stage_ = −0.215, *p* = 7.7 × 10^−6^), post-interference recall (Trial 6, β_stage_ = −0.82, *p* = 4.8 × 10^−16^), 30 min delayed recall (β_stage_ = −0.80, *p* = 3.18 × 10^−14^), and recognition memory trial (β_stage_ = −0.53, *p* = 1.6 × 10^−7^).

Rey Auditory Verbal Learning Test (RAVLT), a measure of verbal episodic memory, showed significant stage-wise decline across all test trials (Figure 5A–I, *p* < 5 × 10^−4^), with repeated learning Trials 1 to 5 revealing preserved initial registration but impaired learning capacity (Figure 5J, β_stage_: −0.15 to −0.60 from Trials 1 to 5). Further, disease stages were also found to be associated with the number of errors on the interference list (Trial B errors: β_stage_ = 0.173, *p* = 5.2 × 10^−7^), indicating that the patients become more susceptible to interference, meaning they have increasing difficulty maintaining previously learned information when presented with new information. These results show that AD progression is characterized by increasingly impaired ability to benefit from repetition, resist interference, and reduced ability to retain information over time, suggesting a systematic breakdown of memory processes, and are in alignment with past studies [22,23,24].

Similarly, logical memory test performance showed significant declines with advancing disease stages (Figure S6), especially in delayed recall (β_stage_ = −0.8, *p* = 4.3 × 10^−16^).

### 2.4. Visuospatial Processing, Motor Planning, and Executive Function

**Figure 6:**
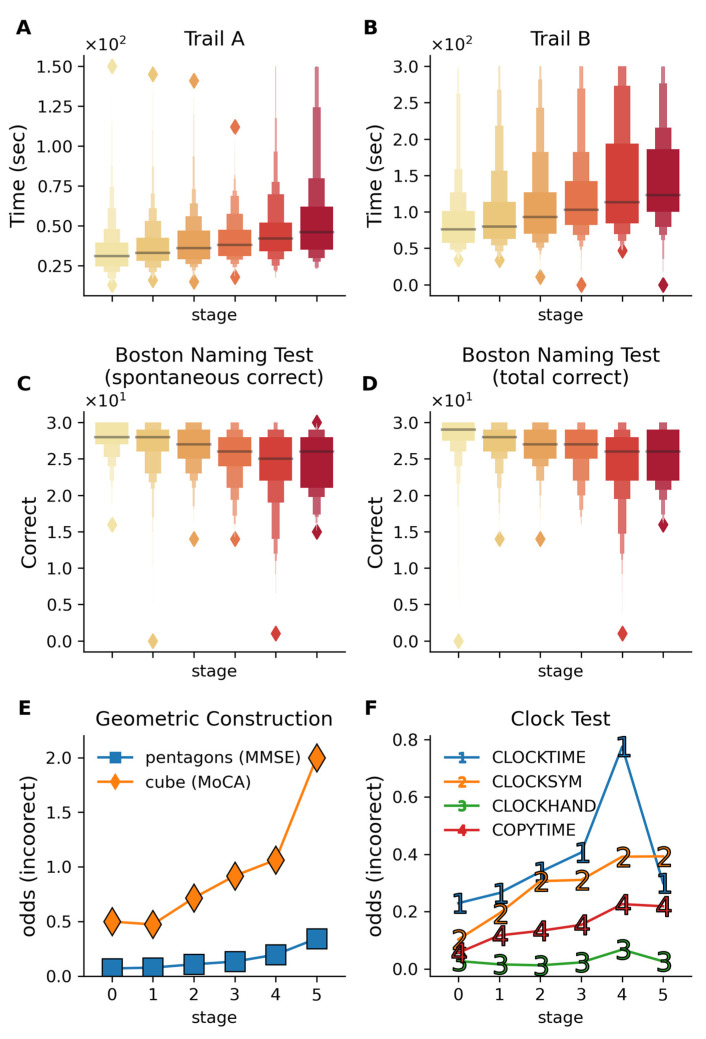
Cognitive performance across multiple visuospatial and executive function tests across disease stages. Color gradient from light to dark in (**A**–**D**) represents increasing disease stages. (**A**,**B**) Trail making test completion times for Trails A and B show progressive slowing in completion time with advancing stages. Trail B (β_stage_ = 10.57, *p* = 2.0 × 10^−8^) shows a stronger decline with disease stage compared to Trail A (β_stage_ = 2.98, *p* = 9.9 × 10^−8^), reflecting the additional cognitive burden in the Trail B test, which requires the subjects to alternatively switch between letters and numbers. These results reflect the deteriorating visual attention, processing speed, and cognitive flexibility with increasing disease stage. (**C**,**D**) The Boston Naming Test demonstrates a decline in visual recognition and confrontational naming ability, with both spontaneous (*p* = 3.5 × 10^−7^) and total correct responses (*p* = 3.3 × 10^−7^) declining with disease stage. (**E**) The odds of incorrect performance on geometric construction tasks increase with disease stages (evaluated via cube drawing on MoCA and pentagon drawing on MMSE). (**F**) Odds of incorrect responses on the clock drawing components increase with disease stages, indicating progressive impairment in visuospatial processing and motor planning. CLOCKTIME—correct times drawn; CLOCKSYM—clock numbers are symmetrically placed; CLOCKHAND—presence of two hands; COPYTIME—correct time shown in the copy condition of the test. All analyses are adjusted for age, gender, education, APOE ε4 alleles, and subtypes.

Cognitive tests for visuospatial processing, motor planning, visual recognition, and executive function were analyzed across disease stages (Figure 6). Linear regression assessed outcomes on the trail making and Boston naming tests, while logistic models were used for binary outcomes (geometric construction and clock drawing), adjusting for demographics, APOE ε4 alleles, and subtype.

#### 2.4.1. Trail Making Test (Processing Speed, Executive Control)

Trail making test performance declined with disease stage (Figure 6A,B), with Trail B showing greater impairment with advancing stage (β_stage_ = 10.57, *p* = 2.0 × 10^−8^) compared to Trail A (β_stage_ = 2.98, *p* = 9.9 × 10^−8^). Trail B showed increased omission (β_stage_ = 0.21 for Trail B vs. β_stage_ = 0.033 for Trail A) and commission errors (β_stage_ = 0.11 for Trail B vs. β_stage_ = 0.02 for Trail A), reflecting its higher executive demand. This aligns with previous findings on Trail B’s utility in predicting progression from MCI to AD [25,26] and further shows the increased rate of both omission and commission errors with advancing stage in Trail B vs. Trail A.

#### 2.4.2. Boston Naming Test (Visual Recognition, Confrontational Naming)

Boston Naming Test scores declined with disease progression (Figure 6C,D), with significant reductions in spontaneous (β_stage_ = −0.61, *p* = 3.3 × 10^−8^) and total responses (β_stage_ = −0.55, *p* = 3.58 × 10^−7^). These results corroborate associations between naming ability and dementia progression [27,28].

#### 2.4.3. Geometric Construction (Visuoconstructional Skills)

Cube copying (β_stage_ = −0.185, *p* = 0.02) and pentagon drawing (β_stage_ = −0.33, *p* = 5.7 × 10^−4^) demonstrated stage-dependent decline (Figure 6E), consistent with prior studies linking these tasks to MCI progression and treatment monitoring [29,30].

#### 2.4.4. Clock Drawing (Visuoconstructional Skills and Executive Function)

Clock drawing scores declined across stages, with command condition impairment exceeding copying (Figure 6F). Errors in symmetry (β_stage_ = −0.29, *p* = 1.1 × 10^−4^) and time setting (β_stage_ = −0.22, *p* = 8 × 10^−4^) aligned with known visuospatial deficits in AD [31,32].

### 2.5. Verbal Fluency and Semantic Memory

Category fluency tests revealed stage-dependent decline (Figure S7). Total correct responses for animal naming (*p* = 2.5 × 10^−9^, β_stage_ = −0.83) were more impaired than vegetable naming (*p* = 2.8 × 10^−3^, β_stage_ = −0.50), suggesting differential vulnerability of semantic categories [33]. Error rates (perseverations, intrusions) showed no significant association with disease stage, suggesting that the decline in categorical fluency manifests primarily as reduced production rather than increased error rates.

### 2.6. Premorbid Verbal Ability

Analysis of the American National Adult Reading Test (ANART), which measures premorbid verbal ability, showed no significant association with disease stage (*p* > 0.05, Figure S8). This lack of correlation serves as a negative control, validating that the staging framework appropriately does not detect a decline in abilities known to remain stable during disease progression [34].

### 2.7. Daily Function, Self-Awareness, and Well-Being

#### 2.7.1. Activities of Daily Living and Self-Awareness

Study partner-reported measures of functional activity and daily living skills—everyday cognition (Ecog-SP) and functional activity questionnaire (FAQ)—showed significant associations with disease stage (Ecog-SP *p* = 7 × 10^−5^, FAQ *p* = 4.5 × 10^−6^, Figure S9), while patient self-reports (ECog-Pt) did not (*p* = 0.08). Subdomain analysis of the Ecog-SP score (Figure S10) revealed declines in memory, language, visuospatial functioning, planning, organizing, and divided attention (*p* < 0.05 in each case). FAQ scores showed impairments in eight of the ten subdomains with advancing disease stages, including memory, shopping, and medication management, aligning with previous findings on functional decline [35,36].

In contrast, patient self-reported ratings on the everyday cognition scale (ECog-Pt) showed no significant association with disease stage, with only the visuospatial subdomain demonstrating a significant decline (Figure S10). Patient and study partner assessments differed significantly, indicating patients’ declining ability to self-report functional changes, aligning with findings in previous work [37].

#### 2.7.2. Mood and Life Satisfaction

The geriatric depression scale (Figure S11) indicated declining life satisfaction (β_stage_ = −0.36, *p* = 0.002) and reduced activity engagement (β_stage_ = −0.31, *p* = 3 × 10^−4^) with advancing stages. These trends support links between depressive symptoms and cognitive decline.

## 3. Discussion

This study presents a comprehensive evaluation of a scalable disease progression model for Alzheimer’s disease, leveraging high-dimensional neuroanatomical data. The findings offer new insights into the biological and clinical relevance of subtype and stage stratification, with implications for prognosis, risk stratification, and precision medicine. Below, we discuss the key clinical contributions, validation results, and potential applications of the s-SuStaIn framework, as well as limitations and future directions.

### 3.1. Prognostic Utility of a Data-Driven Disease Staging Framework

This study demonstrates the clinical utility of a data-driven disease staging framework, derived from neuroanatomical features using s-SuStaIn [15], for predicting progression from mild cognitive impairment (MCI) to Alzheimer’s disease (AD). The model significantly outperformed traditional risk assessment methods based on demographic and genetic factors (C-index = 0.73 vs. 0.62; Figure 1). This prognostic advantage underscores the value of disease staging and subtyping that captures heterogeneity beyond conventional risk markers.

### 3.2. Novel Clinical Contributions and Model Scalability

This work represents the first large-scale application of s-SuStaIn to a comprehensive neuroanatomical dataset encompassing 118 brain regions. Previous SuStaIn implementations have relied on limited biomarker sets and user-curated features. In contrast, this study demonstrates that s-SuStaIn can scale to high-dimensional neuroimaging data while uncovering disease heterogeneity across previously understudied regions. This scalability enabled the detection of significant subtype-specific effects (Figure 1) not observed when using a reduced feature set (Table S1), highlighting the model’s ability to retain biological interpretability while expanding dimensionality.

Importantly, the framework relies solely on widely available structural MRI data, which enhances its clinical feasibility relative to methods requiring expensive molecular biomarkers.

### 3.3. Validation Across Independent Modalities and Cohorts

Study results were validated in the largest publicly available multi-center longitudinal AD dataset (ADNI), involving 808 MCI subjects not used during training. Stages and subtypes generalized well to unseen data and were associated with independent biomarkers and cognitive measures. The model’s biological and clinical relevance was supported by robust associations with multiple independent measures, including the following:

- **Biomarkers:** Subtype-specific and stage-dependent changes in CSF Aβ_42_ and FDG-PET metabolism aligned with known markers of AD progression (Figure 2). Subtype 1 showed higher CSF Aβ_42_ and FDG-PET metabolism alongside reduced risk of progression, consistent with prior findings [38,39,40,41].
- **Cognitive Performance:** Stage-dependent decline was observed in global cognitive scores (Figure 4). Specific impairments in learning, memory (Figure 5), executive function, and semantic memory (Figure 6 and Figure S7) were consistent with hallmark patterns of AD. Notably, category fluency decline occurred primarily via reduced production rather than increased errors, with sex-specific effects evident in the vegetable category only.
- **Functional Assessments:** Study partner-reported functional decline was more strongly associated with disease stage than self-reported measures (Figure 4), a pattern consistent with prior studies [42,43].

### 3.4. Subtype-Specific Prognosis and Implications for Clinical Trials

Significant differences in clinical progression were observed across subtypes. Subtype 2 exhibited the highest risk of conversion to AD (HR = 2.50), while Subtype 1 had the slowest progression and most favorable biomarker profile. Subtypes also differed in symptom onset age (Figure S2), indicating distinct progression trajectories. Table A2 shows differences in medical history across the subtypes. Subtypes reveal distinct patterns that both align with and extend our understanding of AD neuropathological progression:

- **Subtype 1 (Subcortical-First Pattern):** This subtype shows early involvement of subcortical structures (caudate, pallidum) and ventricular systems before affecting classical AD regions like the hippocampus and entorhinal cortex (which change in the final stage). This pattern suggests a vascular or mixed pathology variant, where subcortical changes may reflect cerebrovascular disease or different tau/amyloid deposition patterns. The late involvement of medial temporal structures aligns with better-preserved memory function observed in this subtype.
- **Subtype 2 (Executive–Cortical Pattern):** Early frontal and posterior cingulate involvement followed by classic medial temporal progression mirrors the “outside–in” cortical pattern described in atypical AD variants. The late-stage hippocampal/entorhinal changes suggest this represents an executive-predominant phenotype where tau pathology may follow different cortical networks before reaching classical memory circuits. This aligns with reports of AD patients presenting with executive dysfunction rather than memory impairment.
- **Subtype 3 (Disconnection Pattern):** The early corpus callosum and bilateral thalamic involvement reflects white matter tract vulnerability and connectivity hub disruption. This pattern suggests tau spreads via trans-synaptic mechanisms along major white matter pathways, consistent with the recent understanding of tau propagation through neural networks. The relatively late hippocampal involvement indicates preserved memory networks until advanced stages.
- **Subtype 4 (Frontal–Executive Pattern):** Extensive early frontal involvement with very late medial temporal changes represents the most atypical progression pattern. This may reflect primary age-related tauopathy (PART) or suspected non-Alzheimer pathophysiology (SNAP), where tau pathology predominantly affects frontal networks. The pattern resembles behavioral variant frontotemporal dementia in early stages, highlighting diagnostic challenges in atypical AD presentations.

Our data-driven approach identifies network-specific vulnerability patterns that may reflect different underlying pathophysiological mechanisms, genetic susceptibilities (APOE4 effects), or mixed pathologies. The superior prognostic performance (C-index = 0.73) suggests these volume-based staging patterns capture clinically meaningful biological heterogeneity beyond traditional neuropathological staging systems. Table A1 summarizes important differences across subtypes and stages (CSF Aβ_42_, FDG, volumetrics, neuropsychological).

These findings support the framework’s potential to stratify patients by risk and therapeutic responsiveness. Subtype–stage stratification may uncover treatment effects otherwise masked in pooled analyses. For instance, prior work using SuStaIn identified meaningful heterogeneity in 42% of A4 trial participants, with distinct cognitive trajectories across subtypes [42]. Our s-SuStaIn implementation extends this approach by incorporating nearly ten times more neuroanatomical features (118 vs. 13), providing a more detailed representation of disease heterogeneity. This enhanced granularity may improve patient selection for trials and enable precision monitoring strategies in clinical practice.

### 3.5. Translational Value and Feasibility

The ability to stratify patients using only structural MRI makes this framework highly accessible for clinical use. By capturing rich anatomical changes and demonstrating strong associations with progression risk, s-SuStaIn offers a cost-effective tool for personalized patient counseling, risk stratification for clinical trial planning, the early detection of high-risk cases, and stratified clinical trial design. Additional subtype- and stage-specific findings are presented in the Supplemental Materials (Table S1 and Figures S1–S11).

### 3.6. Limitations and Future Directions

While s-SuStaIn advances the scalability and interpretability of subtype models, it retains certain limitations of unsupervised modeling. Specifically, it does not directly map subtypes to established clinical phenotypes. Further validation in external cohorts is ongoing, but generalizability may be influenced by variations in cohort characteristics (e.g., genetics and lifestyle) and clinical workflows (e.g., diagnosis and imaging protocols). Future work will focus on extending the model to additional modalities (e.g., Tau PET, CSF, and plasma biomarkers); refining subtype-to-clinical mapping methods; applying the model to large-scale biobanks; and evaluating subtype-specific therapeutic response. By integrating multimodal data, s-SuStaIn could offer a comprehensive disease characterization framework for advancing personalized care in Alzheimer’s disease.

## 4. Materials and Methods

### 4.1. Study Data

The study utilized data from the Alzheimer’s Disease Prediction of Longitudinal Evolution (TADPOLE) challenge [43]. Neuroimaging features, including brain region volumes, cortical thicknesses, and surface areas, were obtained from the Laboratory of Neuroimaging (LONI) data archive (https://adni.loni.usc.edu/), specifically from the dfMri_D12.csv file (accessed on 25 August 2024).

The initial dataset comprised 123 neuroimaging features. Data preprocessing involved the removal of five features: four (Left-WM-hypointensities, Right-WM-hypointensities, Left-non-WM-hypointensities, Right-non-WM-hypointensities) due to zero variance, and one (5th ventricle volume) due to a heavy-tailed distribution that precluded fitting of a two-component mixture model as described in [44]. The final preprocessed dataset included 118 features.

The study population consisted of three mutually exclusive diagnostic groups from the ADNI database (ADNI-1/2/GO): cognitively normal controls (CN, *n* = 504), Alzheimer’s disease-diagnosed participants (AD, *n* = 346), and individuals with mild cognitive impairment (MCI, *n* = 808). Additional clinical information was sourced from multiple ADNI tables: diagnostic classifications, CSF marker levels (Aβ42), FDG-PET measurements, and global cognitive function (MMSE, MoCA, ADAS, etc.) were extracted from the ADNIMERGE table, and other neuropsychological assessments (GDS, FAQ, and everyday cognition) were obtained from their respective specialized tables. The cognitive tests broadly belonged to two categories—general screening tests such as ADAS, MoCA, and MMSE (Figure 4), which provide a broad assessment of multiple cognitive domains, and more specific cognitive tests, which tested specific cognitive domains such as episodic memory (RAVLT, Figure 5), visual recognition (Boston Naming Test, Figure 6), visuoconstructional skills (clock making test, cube drawing test, Figure 5), semantic memory (category fluency test, Figure S7), and functional assessments (Figures S9 and S10). By considering these two categories of cognitive tests and showing their association with inferred disease stages, our work provides evidence that the disease progression model associates not only with general measures of cognitive health useful for screening but also with specific cognitive domains. Finally, a third category of a negative control test was also used in the validation, ANART (American National Adult Reading Test), which is a useful test for premorbid verbal ability and serves as a negative control test for cognitive decline.

### 4.2. Disease Progression Modeling

The study employed s-SuStaIn (scaled Subtype and Stage Inference) [15], a variant of the event-based model (EBM) and a scalable version of the SuStaIn algorithm [14]. While the SuStaIn algorithm has been applied to study progression in Alzheimer’s, frontotemporal dementia, and multiple sclerosis [14,17] using data from a limited number of brain regions, this study presents the first major application of its scalable variant (s-SuStaIn) to a large set of brain-wide anatomical features (d = 118) in AD. The inferred disease subtypes and stages from this analysis were then used to stratify conversion risk to AD in MCI subjects and uncover progression patterns across diverse biomarkers such as brain volumetrics, CSF, FDG-PET, and neuropsychological assessments.

The training dataset comprised single visits (earliest available) from cognitively normal controls (CN, *n* = 504) and AD-diagnosed patients (*n* = 346), resulting in a total training cohort of 850 participants. Additional features such as neuropsychological scores, CSF biomarkers (Aβ42), and PET imaging markers were used in model validation but not during training.

The trained model was subsequently applied to an independent validation cohort consisting of participants with mild cognitive impairment (MCI, *n* = 808). There was no overlap between subjects in the training and validation cohorts, ensuring the independent validation of the model’s performance.

The disease progression trajectory for each subtype was derived by clustering 118 neuroanatomical features into five clusters, yielding six distinct disease stages (0 through 5). A minimum cluster size of eight was implemented to optimize the balance between granularity and model stability. The maximum number of disease subtypes was constrained to four, informed by the prior literature, established clinical staging frameworks, and model likelihoods. This modeling architecture resulted in a six-stage progression, aligning with established clinical frameworks such as Braak staging, which also comprises six stages.

In contrast to earlier SuStaIn models that do not utilize biomarker clustering and often produce as many stages as input features, this design limits the number of stages to enhance interpretability and clinical relevance. Excessively granular stage outputs may obscure meaningful clinical distinctions. Therefore, reducing the number of stages while maintaining disease progression fidelity improves the potential for clinical application.

With respect to the number of subtypes, prior systematic reviews have consistently indicated the presence of four distinct subtypes in Alzheimer’s disease [45]. Model likelihoods were compared across configurations with two, three, and four subtypes using the s-SuStaIn framework. The configuration with four subtypes yielded the highest data likelihood, providing the empirical basis for selecting four subtypes in the final model.

Model uncertainty was characterized using Markov chain Monte Carlo (MCMC) sampling. For each subtype level, 2 × 10^6^ MCMC samples were generated, with the first 1 × 10^6^ iterations discarded as burn-in. Subject subtyping and staging were performed using 1 × 10^3^ samples drawn from the remaining 1 × 10^6^ MCMC samples, following previously published methodology [14,46,47].

### 4.3. Statistical Analysis

Relationships between inferred disease stages and multiple outcome measures were assessed using regression models. For continuous outcomes such as regional brain volumes, CSF biomarkers (Aβ42 levels), FDG-PET markers, and cognitive test scores, linear regression models were employed. Binary outcomes were analyzed using logistic regression.

In all models, disease stages and subtypes served as the primary explanatory variables. Models were adjusted for potential confounding factors, including demographic variables (age, gender, and education years) and genetic risk (number of APOE4 alleles). Significant associations were established based on two criteria: the individual coefficients (for stage or subtype) demonstrating significant effect sizes (*p* < 0.05) and the overall model showing statistical significance (F-statistic *p* < 0.05 for linear regression or likelihood ratio test *p* < 0.05 for logistic regression).

Progression from MCI to AD was analyzed using a Cox proportional hazards (CPH) model in the validation cohort of MCI subjects (*n* = 808, of which 671 had longitudinal data available). Two models were compared: a baseline model incorporating only demographic variables (age, gender, and education years) and APOE4 status, and a full model that additionally included s-SuStaIn-derived measures (disease stage and subtype). Disease subtypes, being categorical, were encoded as one-hot vectors with the most prevalent subtype serving as the reference group. Model comparison was performed using the concordance index and the Akaike Information Criterion (AIC). The prognostic value of the subtypes and stages was evaluated by the significance of their effect sizes in predicting conversion from MCI to AD.

## Data Availability

We use Alzheimer’s Disease Neuroimaging Initiative (ADNI) data made available as a part of the TADPOLE challenge [43]. It was downloaded via the Laboratory Of Neuroimaging data archive at https://adni.loni.usc.edu/ (accessed 25 August 2024).

## Supplementary Materials

The following supporting information can be downloaded at https://www.mdpi.com/article/10.3390/ijms26125514/s1.

## Abbreviations

The following abbreviations are used in this manuscript:

ADAS	Alzheimer’s Disease Assessment Scale
AIC	Akaike Information Criterion
ANART	American National Adult Reading Test
CDR-SB	Clinical Dementia Rating Sum of Boxes
C-Index	Concordance Index
Cox PH	Cox Proportional Hazards Model
CSF	Cerebrospinal Fluid
Ecog-Pt	Everyday Cognition (Patient Score)
Ecog-SP	Everyday Cognition (Study Partner Score)
FAQ	Functional Activities Questionnaire
FDG	Fluorodeoxyglucose
MCI	Mild Cognitive Impairment
MoCA	Montreal Cognitive Assessment
MMSE	Mini-Mental State Examination
PACC	Preclinical Alzheimer’s Cognitive Composite
PET	Positron Emission Tomography
RAVLT	Rey’s Auditory Verbal Learning Test
s-SuStaIn	Scaling Subtype and Stage Inference
SuStaIn	Subtype and Stage Inference

## Appendix A

### Appendix A.1

**Table A1:** Summary of key biomarkers that showed differences across stages and subtypes.

**Biomarker**	**Differences Across Stages**	**Difference Across Subtypes**
CSF Aβ42	Yes	Yes (Subtype 1 vs. Subtype 2)
FDG-PET	Yes	Yes (Subtype 1 vs. Subtype 2)
Brain Volumetrics		
Entorhinal Cortex	Yes	No
Fusiform	Yes	Yes (Subtype 1 vs. Subtype 2)
Hippocampus	Yes	Yes (Subtype 1 vs. Subtype 4)
Middle Temporal	Yes	Yes (Subtype 1 vs. Subtype 2)
Ventricles	Yes	Yes (Subtype 1 vs. Subtype 2) (Subtype 1 vs. Subtype 3) (Subtype 1 vs. Subtype 4)
Whole Brain	Yes	Yes (Subtype 1 vs. Subtype 2)
Neurocognitive Assessments		
ADAS11	Yes	Yes (Subtype 1 vs. Subtype 4)
ADAS13	Yes	Yes (Subtype 1 vs. Subtype 2)
MMSE	Yes	No
MoCA	Yes	No
mPACCTrailB	Yes	No
CDRSB	Yes	Yes (Subtype 1 vs. Subtype 2)

### Appendix A.2

**Table A2:** Comorbidities and medical history across inferred subtypes. The numbers represent the count of individual with the associated medical history, and the percentage represents the prevalence in the subtype group. All subjects in the study (CN/MCI/AD) are used here. * (*p* < 0.05), ** (*p* < 0.005).

**Characteristics**	**Subtype_1_**	**Subtype_2_**	**Subtype_3_**	**Subtype_4_**	***p*** **-Value (*χ*^2^)**
Psychiatric	280 (35.4%)	105 (30.9%)	87 (32.7%)	87 (33.1%)	0.5
Neurological (non-AD)	227 (28.7%)	107 (31.5%)	72 (27.0%)	79 (30.0%)	0.64
Head, Eyes, Ears, Nose and Throat	471 (59.6%)	223 (65.8%)	173 (65.0%)	173 (65.8%)	0.1
Cardiovascular	520 (35.4%)	243 (30.9%)	184 (32.7%)	172 (33.1%)	0.2
Respiratory	167 (21.1%)	74 (21.8%)	56 (21.1%)	59 (22.4%)	0.9
Hepatic	20 (2.53%)	17 (5.0%)	13 (4.9%)	12 (4.6%)	0.1
Dermatologic-Connective Tissue	225 (28.5%)	128 (37.8%)	81 (30.5%)	86 (32.69%)	0.02 *
Musculoskeletal	534 (67.6%)	215 (63.4%)	182 (68.4%)	174 (66.1%)	0.51
Endocrine-Metabolic	349 (44.2%)	122 (36.0%)	113 (42.5%)	121 (46.0%)	0.043 *
Gastrointestinal	361 (45.7%)	145 (42.8%)	123 (46.2%)	113 (43.0%)	0.7
Hematopoietic-Lymphatic	66 (8.4%)	28 (8.3%)	25 (9.4%)	29 (11.0%)	0.057
Renal-Genitourinary	320 (40.5%)	162 (47.8%)	105 (39.5%)	126 (47.9%)	0.027 *
Allergies or Drug Sensitivities	332 (42.0%)	136 (40.1%)	112 (42.1%)	115 (43.7%)	0.85
Alcohol Abuse	22 (2.8%)	16 (4.7%)	21 (7.9%)	13 (4.9%)	0.004 *
Drug Abuse	8 (1.0%)	3 (0.8%)	1 (0.3%)	1 (0.4%)	0.63
Smoking	280 (35.4%)	143 (42.2%)	132 (49.6%)	96 (36.5%)	0.0003 **
Malignancy	154 (19.5%)	86 (25.4%)	72 (27.1%)	73 (27.75%)	0.0064 *
Skin and Appendages	118 (14.9%)	78 (23.0%)	38 (14.3%)	48 (18.3%)	0.005 *
History of Hypertension	369 (46.7%)	180 (53.1%)	139 (52.3%)	113 (43.0%)	0.035 *
**Hachinski Score**					
0	391	147	117	139	
1	351	166	133	108	
2	33	12	4	9	
3	13	12	12	6	
4	2	2	0	1	0.048
