# Supplementary material for: Comprehensive Anatomical Staging Predicts Clinical Progression in Mild Cognitive Impairment: A Data-Driven Approach
Source: Int J Mol Sci. 2025 Jun 9;26(12):5514. doi: 10.3390/ijms26125514 (PMC12193007; doi:10.3390/ijms26125514)
Supplement: Supplementary file 1 [file ijms-26-05514-s001.zip › ijms-3645765-supplementary.pdf]

Supplementary Information:

Comprehensive Anatomical Staging Predicts Clinical Progression in Mild Cognitive Impairment: A Data-Driven Approach

Raghav Tandon [1,2], Yajun Mei [3], James J. Lah [4] and Cassie S. Mitchell [1,2,*]

[1] Laboratory for Pathology Dynamics, Department of Biomedical Engineering, Georgia Institute of Technology and Emory University School of Medicine, Atlanta, GA 30332, USA
[2] Center for Machine Learning, Georgia Institute of Technology, Atlanta, GA 30332, USA
[3] Department of Biostatistics, School of Global Public Health, New York University, New York, NY 10003, USA
[4] Alzheimer's Disease Research Center, Department of Neurology, Emory University School of Medicine, Atlanta, GA 30329, USA
* Correspondence: cassie.mitchell@bme.gatech.edu

Table S1: s-SuStaIn is applied to a varying number of neuroimaging features. These feature subsets were obtained in two different ways. Cases 1-3: features were selected based on their perceived importance from a clinical standpoint and use in previous literature. Cases 4-7: features were selected based on statistically significant differences between the CN and AD populations in the data. The test statistic and p-values were obtained from the Mann-Whitney U test and corrected using the Bonferroni correction. Finally in case 8, all 118 features in the data were utilized without excluding any feature. Only in the final case when all features are utilized, each subtype shows a statistically significant effect in predicting MCI to AD progression in a Cox proportional hazards model.

Case	Brain regions selected
**1**	Hippocampus, Parahippocampus, Entorhinal cortex, Amygdala, Cingulate, Frontal cortex, Occipital, Ventricles (d=34)
**2**	Hippocampus, Parahippocampus, Entorhinal cortex, Amygdala, Cingulate, Frontal cortex, Occipital, Ventricles, Temporal cortex, Insula (d=46)
**3**	Hippocampus, Parahippocampus, Entorhinal cortex, Amygdala, Cingulate, Frontal cortex, Occipital, Ventricles, Temporal cortex, Insula, Thalamus, Putamen, Pallidum, Caudate, Accumbens, Parietal, Total cranial volume (d=61)
**4**	All brain regions with $p < 10^{-14}$ between CN and AD (d=41)
**5**	All brain regions with $p < 10^{-10}$ between CN and AD (d=51)
**6**	All brain regions with $p < 10^{-6}$ between CN and AD (d=73)
**7**	All brain regions with $p < 10^{-2}$ between CN and AD (d=87)
**8**	All brain regions (d=118)

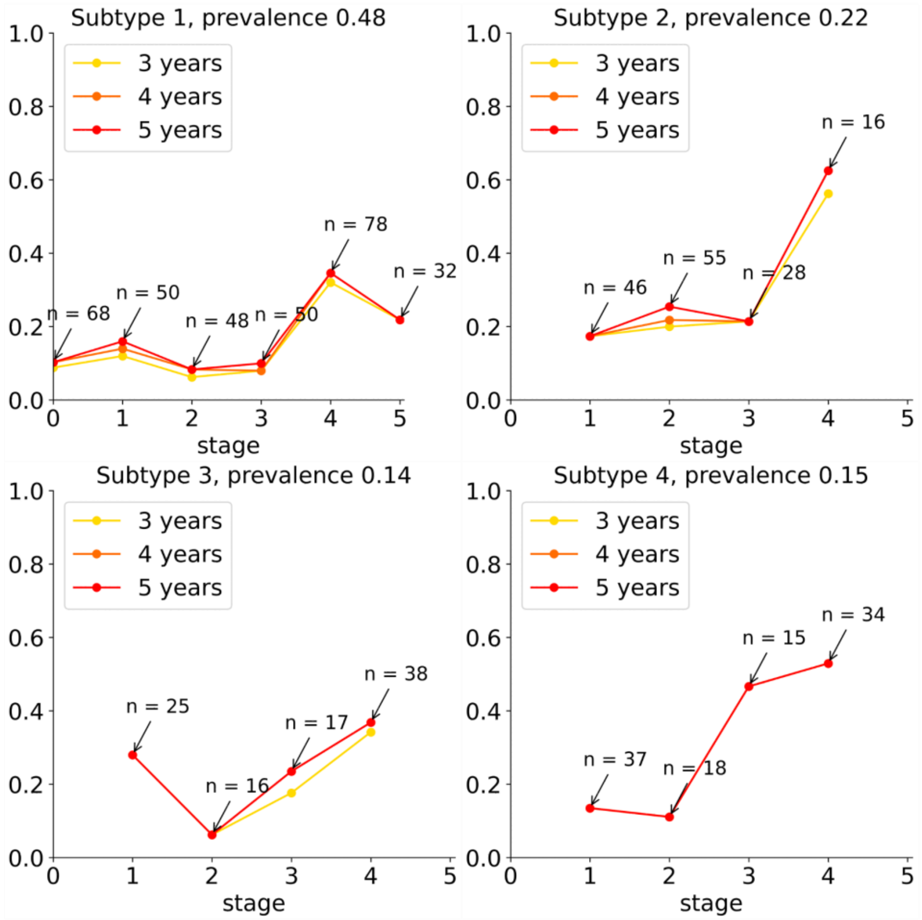

Figure S1: Stage-specific conversion rates from MCI to AD dementia across disease subtypes in the validation cohort. Line plots show the cumulative fraction of MCI subjects converting to AD dementia over 3-year (yellow), 4-year (orange), and 5-year (red) follow-up periods for each disease subtype. Subtype prevalence is indicated in subplot titles, with subtype 1 being most prevalent (0.48) followed by subtype 2 (0.22), subtype 4 (0.15), and subtype 3 (0.14). The number of subjects (n) at each disease stage is annotated. Cox proportional hazards model are fit to each subtype separately and stages are found to have a significant effect sizes in each case (3 year conversion risks) - $subtype_1$ ($\beta = 1.81$, $p = 1.3\text{x}10^{-7}$), $subtype_2$ ($\beta = 1.98$, $p = 6.1\text{x}10^{-4}$), $subtype_3$ ($\beta = 1.67$, $p = 2.2\text{x}10^{-2}$), $subtype_4$ ($\beta = 1.8$, $p = 1.1\text{x}10^{-3}$), while adjusting for demographics and number of APOE ε4 alleles. Conversion patterns vary across subtypes: subtype 1 shows moderate conversion rates whereas subtype 2 demonstrates relatively stable rates until a sharp increase at stage 4.

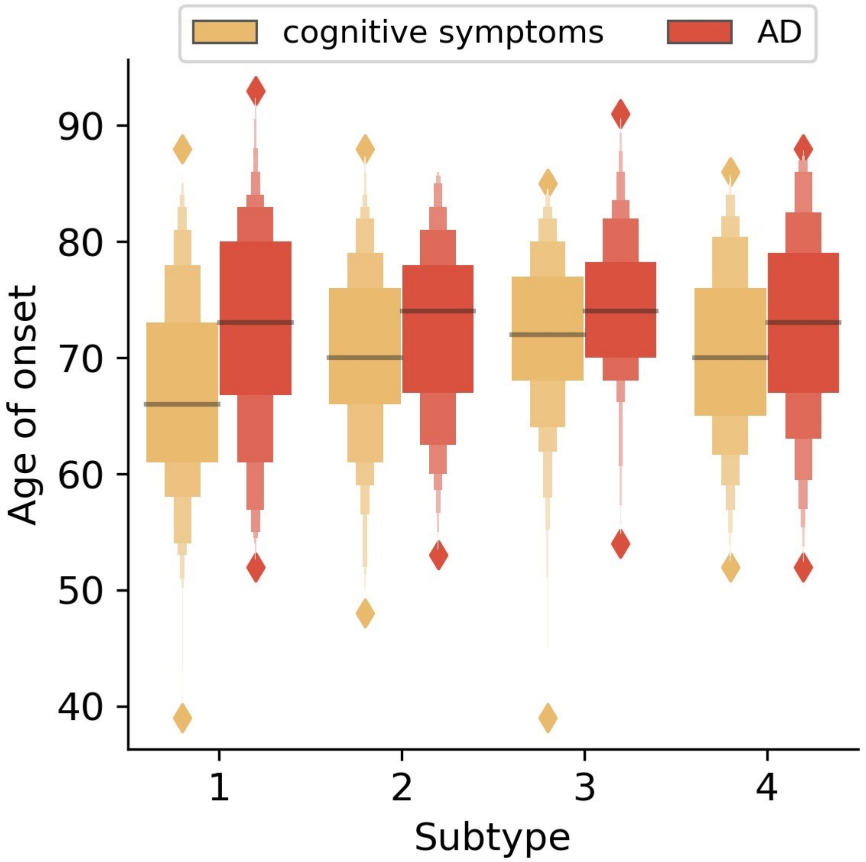

Figure S2: Distribution of age of symptom and disease onset across subtypes in the validation cohort. Box plots show the age of initial cognitive symptoms (yellow) and Alzheimer's Disease (AD) diagnosis (red) for each subtype (1-4), after adjusting for gender, education, and APOE ε4 status. Age of cognitive symptom onset differed significantly across subtypes (Kruskal-Wallis $p < 5\times10^{-8}$), while age at AD diagnosis showed no significant variation between subtypes. Notably, the temporal gap between cognitive symptom onset and AD diagnosis varies across subtypes, potentially reflecting distinct progression patterns.

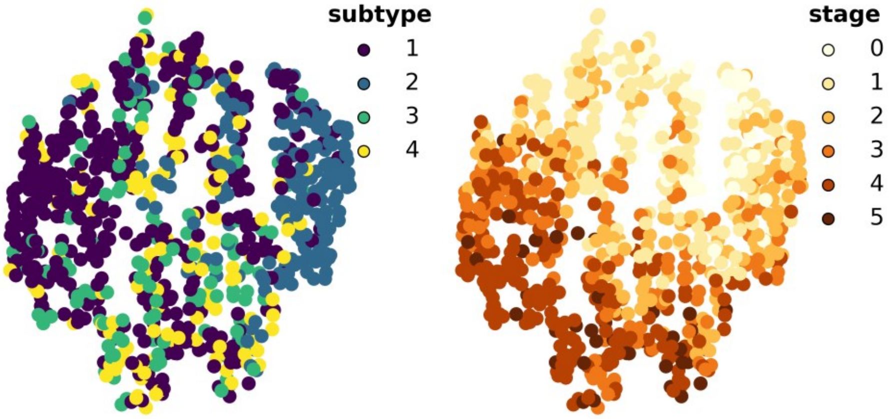

Figure S3: Two-dimensional t-SNE visualization of the neuroanatomical features in MCI patients forming the validation cohort. Left panel shows patient distribution colored by disease subtypes (1-4), revealing both distinct clustering and overlapping patterns among subtypes, with subtype 1 being more prominent in the left regions and subtype 2 displaying more localized clustering in the right regions of the t-SNE projection. Right panel presents the same projection colored by disease stages (0-5, light to dark), demonstrating a continuous progression pattern where earlier stages predominantly appear in the top regions while advanced stages cluster in the bottom regions. The visualization captures the complex relationships between disease subtypes while simultaneously revealing gradual progression trajectories in the high-dimensional data space. Points represent individual MCI subjects, with proximity in the projection space indicating similarity in their disease characteristics.

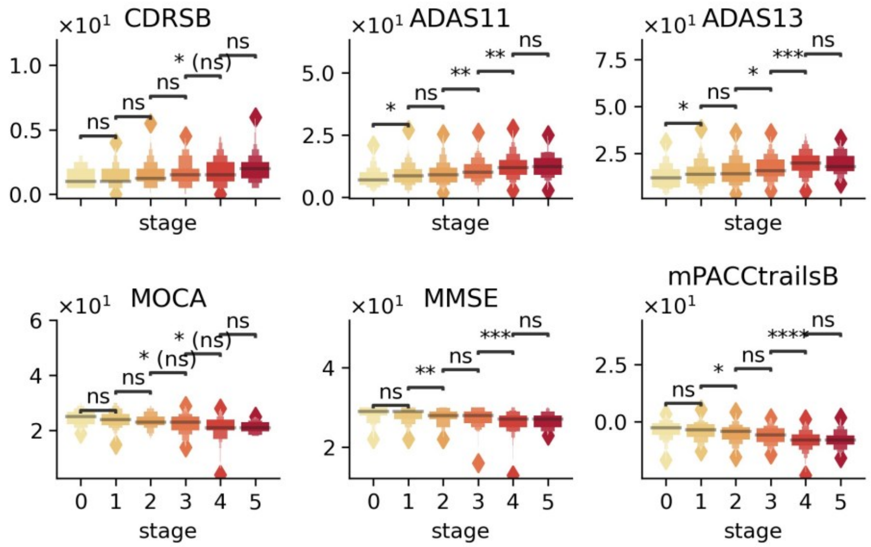

Figure S4: Stage-wise progression of cognitive measures in MCI cohort. Distribution of scores across disease stages (0-5) for global clinical staging (CDR-SB), cognitive assessments (ADAS11, ADAS13, MMSE, MoCA), and composite measure (mPACCtrailsB). Figures show score distributions at each stage, with black horizontal lines indicating medians. Statistical comparisons between consecutive stages are shown (Mann-Whitney test, FDR corrected, *p < 0.05, **p < 0.005, ***p < 0.0005, ns: not significant), adjusted for age, gender, education, APOE ε4 allele count and disease subtypes. Higher scores indicate greater impairment for CDR-SB, ADAS11 and ADAS13, while lower scores indicate greater impairment for MMSE, MoCA, and mPACCtrailsB. ADAS11 and ADAS13 demonstrate particular sensitivity to early-stage transitions (stages 0-1), while MMSE and mPACCtrailsB show sensitivity to later stages (stages 3-4). Color gradient from light to dark represents increasing disease stages.

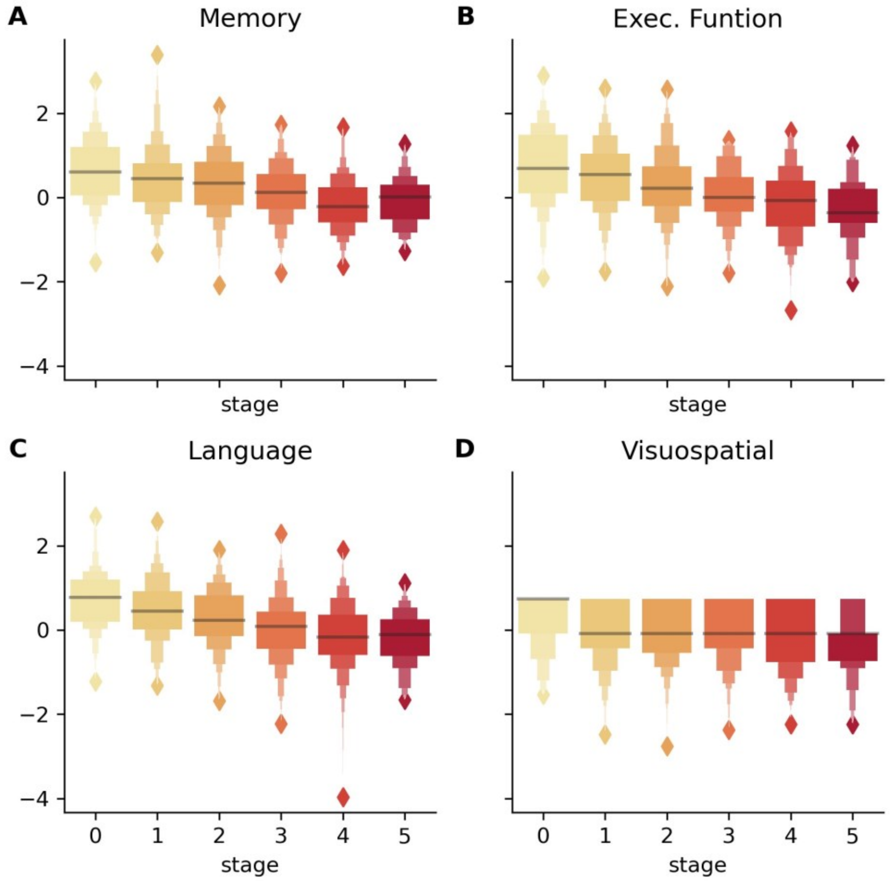

Figure S5: Association between disease stages and cognitive domain scores in the validation cohort. Box plots show the stage-wise progression (stages 0-5) of four cognitive domains derived from the composite framework developed by Crane et. al., 2012 : Memory, Executive Function, Language, and Visuospatial abilities. All scores are standardized and adjusted for age, gender, education, APOE ε4 status, and disease subtypes. Highly significant associations with disease stages were observed across all domains (Memory: $p = 9.72\times10^{-16}$, Executive Function: $p = 5.70\times10^{-13}$, Language: $p = 1.39\times10^{-13}$, Visuospatial: $p = 1.40\times10^{-3}$), with progressively lower scores in advanced stages. Color gradient from light to dark represents increasing disease stages.

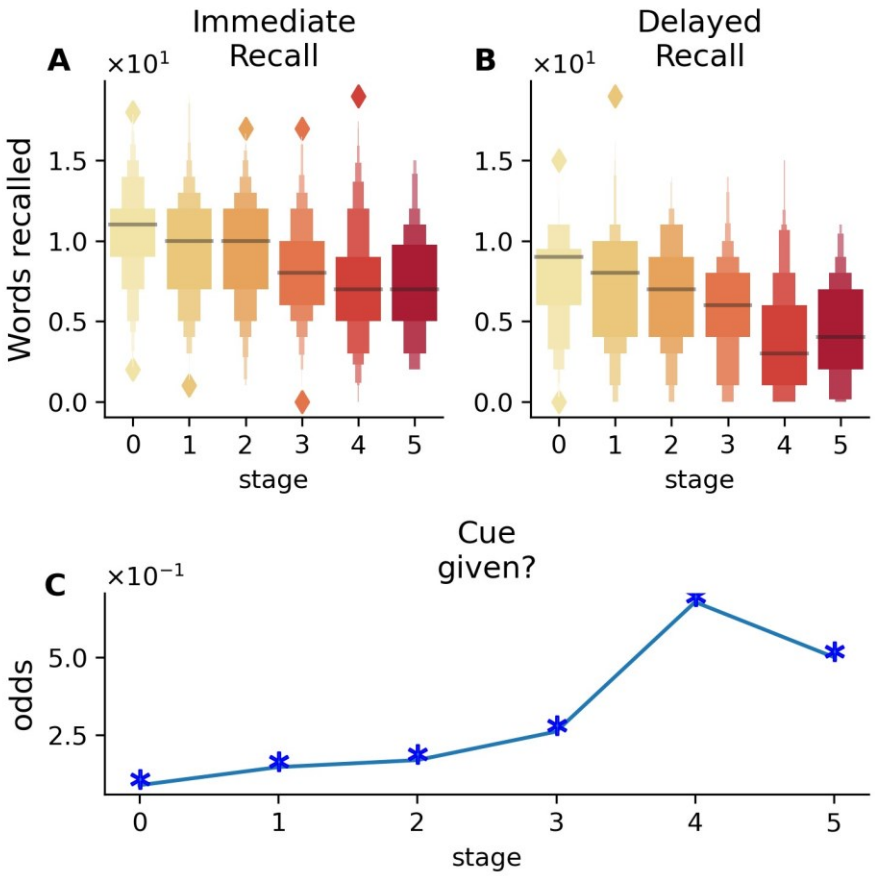

Figure S6: Logical Memory test performance across disease stages. Top panels show distribution of A) immediate and B) delayed story recall scores across stages 0-5, with color gradient from light to dark representing increasing disease stages. Lower panel displays the odds of requiring cues during delayed recall. All analyses adjusted for age, gender, education, APOE ε4 status, and disease subtypes. Significant association with disease stage were observed for immediate recall ($p = 1.6x10^{-10}$), delayed recall ($p = 4.3x10^{-16}$), and whether a cue was provided ($p = 9.9x10^{-8}$).

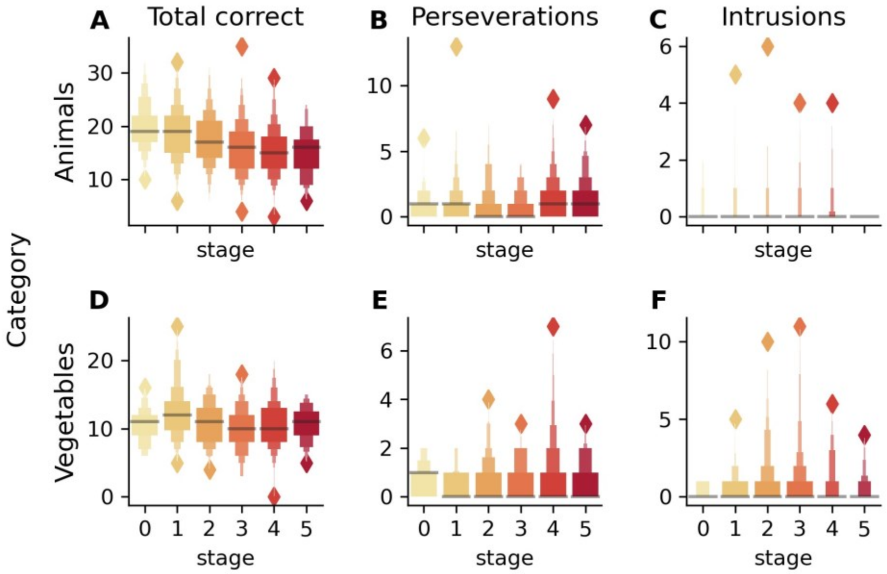

Figure S7: Performance on category fluency tests across disease stages. Analysis of animal (fig, A,B,C) and vegetable (fig. D,E,F) category fluency tests across disease stages (0-5) shows impairment in naming ability with advancing disease stages (fig. A,D) but no significant changes in perseveration (fig. B,E) or intrusion errors (fig. C,F). A stronger decline in total correct responses was observed for animal naming (fig. A) $p = 2.5\times10^{-9}$, $\beta = -0.83$) compared to vegetable naming (fig. B) $p = 2.8\times10^{-3}$, $\beta = -0.50$), while error rates (perseverations and intrusions) remained relatively stable across disease stages ($p > 0.05$).

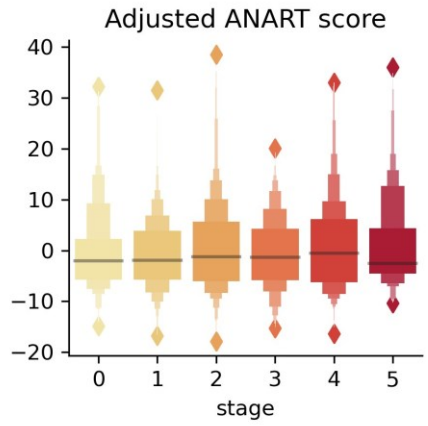

Figure S8: Distribution of adjusted ANART scores across disease stages. No significant association is found between the adjusted scores and the disease stage, supporting the staging framework's specificity in modeling cognitive domain. Color gradient from light to dark represents increasing disease stages.

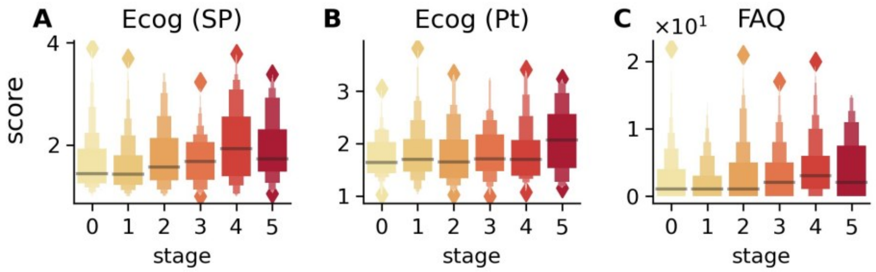

Figure S9: Association between disease stages and functional assessments in the validation cohort. Box plots display the distribution of scores across disease stages (0-5) for three functional measures: Study Partner-reported Everyday Cognition Scale (Ecog-SP), Patient-reported Everyday Cognition Scale (Ecog-Pt), and Functional Activities Questionnaire (FAQ). Higher scores indicate greater functional impairment. Significant associations with disease stages were observed for study partner-reported measures while adjusting for age, gender, education, APOE ε4 alleles, and subtypes (Ecog (SP): $p = 7.6\times10^{-5}$; FAQ: $p = 4.5\times10^{-6}$), while patient self-reported ratings on the Everyday Cognition scale (Ecog-Pt) showed no significant association ($p = 0.08$) with advancements in disease stage. This suggests potential differences in awareness of functional decline between patients and their study partners. Color gradient from light to dark represents increasing disease stages. Associations between scores and disease stages are adjusted for age, gender, education, APOE ε4 status, and disease subtypes.

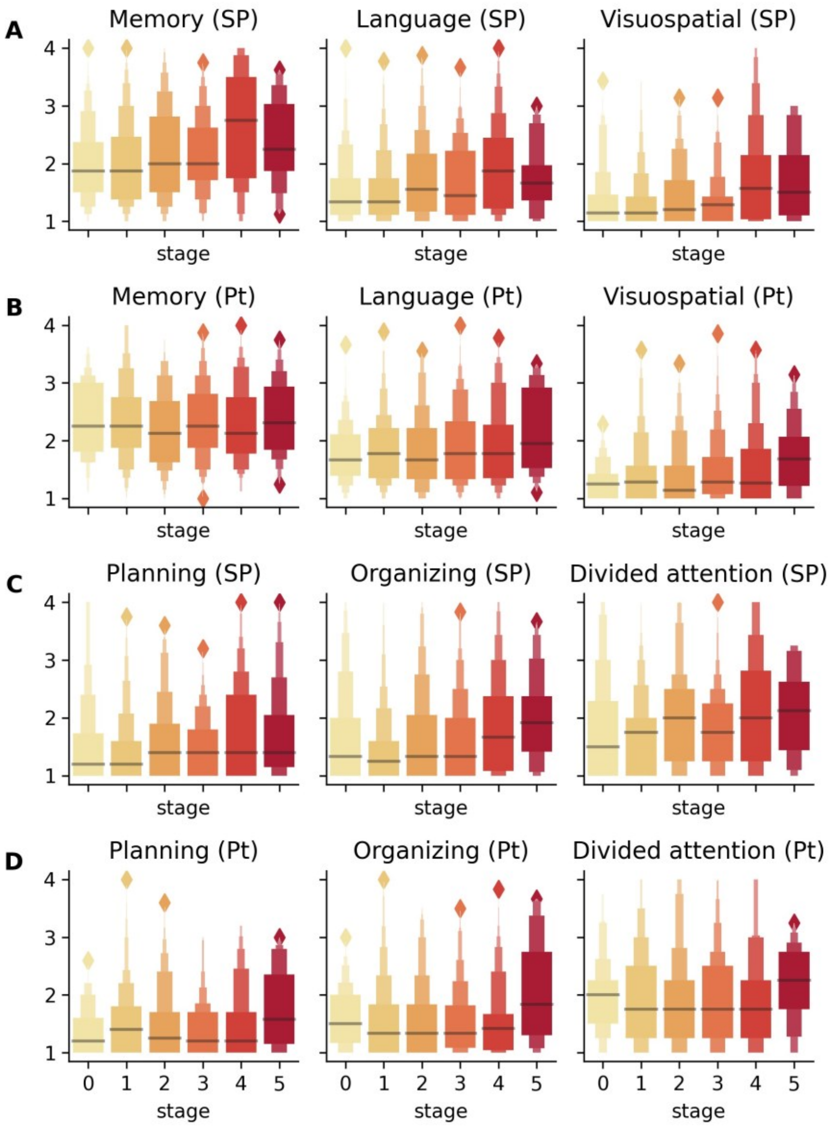

Figure S10: Domain-specific functional assessments across disease stages comparing study partner and patient ratings using the Everyday Cognition (ECog) scale in the validation cohort. Box plots display the distribution of scores across disease stages (0-5) for six cognitive domains: Memory, Language, Visuospatial abilities, Planning, Organizing, and Divided attention. Each domain is rated by both study partners (SP, fig. A,C) and patients (Pt, fig. B,D). Higher scores indicate greater perceived functional impairment. Associations between ratings and disease stages are adjusted for age, gender, education, APOE ε4 status, and disease subtypes. Study partner ratings showed significant associations with disease stage across all cognitive subdomains ($p < 0.05$), while patient self-ratings demonstrated significant association only for visuospatial abilities, suggesting potential differences in awareness of functional decline across cognitive domains. Color gradient from light to dark represents increasing disease stages.

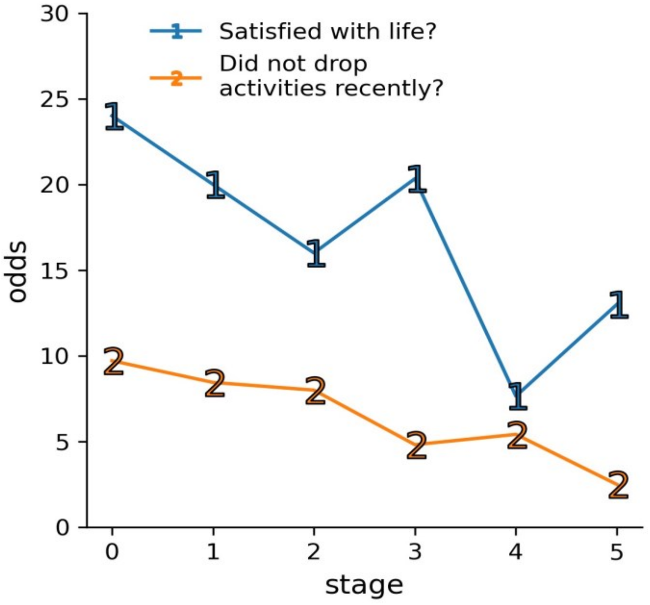

Figure S11: Relationship between disease stages and psychological well-being measures from the Geriatric Depression Scale (GDS). Blue line represents odds of reporting satisfaction with life ($p = 0.002$), while orange line shows odds of maintaining activities and interests ($p = 3\times10^{-4}$) across disease stages 0-5. Associations adjusted for age, gender, education, and APOE ε4 status.
